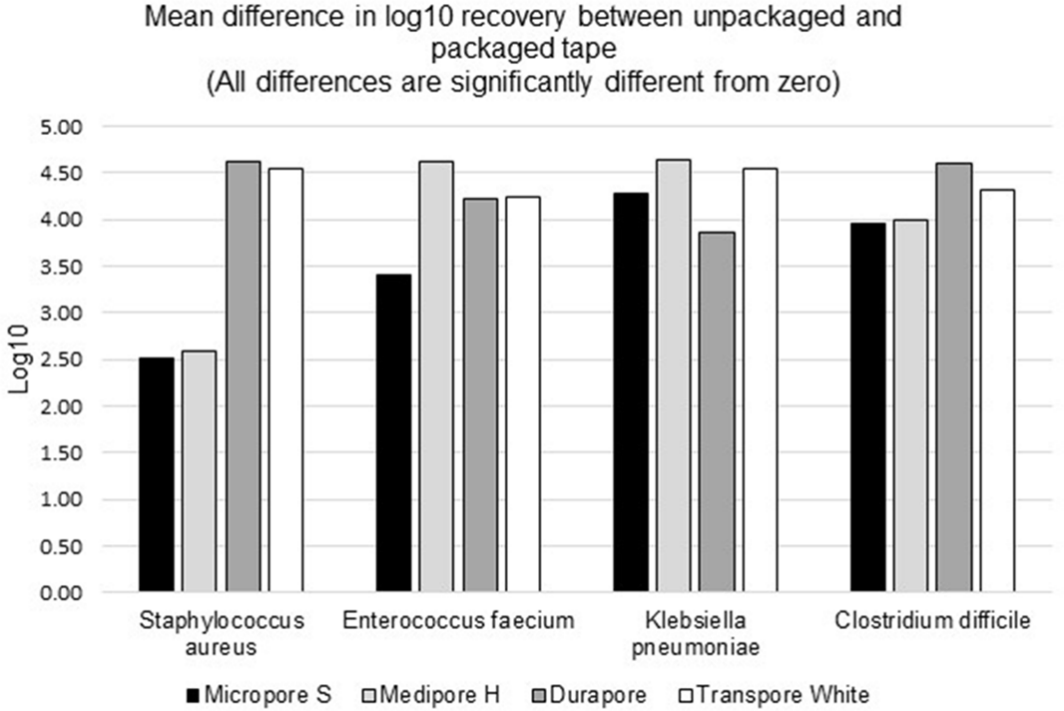# Medical Tape Contamination Study: Effect of Packaging on the Reduction of Cross Contamination

**DOI:** 10.1017/ash.2021.131

**Published:** 2021-07-29

**Authors:** Kheng Vang, Graham Smith, Sara J Pastoor

## Abstract

**Background:** Medical tape is used routinely for a variety of tasks across healthcare settings. The literature contains numerous publications in which common practices around medical tapes have been suspected to lead to infection transmission. Healthcare providers can turn to individually packaged single-patient-use medical tape rolls to help reduce cross-contamination risk by limiting exposure to environmental contaminants, minimizing contact with hospital surfaces and equipment, and minimizing exposure to healthcare workers’ hands and other patients. **Methods:** We evaluated the effect of individually packaged tape on cross contamination using a controlled laboratory assay. Ceramic tiles were inoculated with microorganisms evenly spread across the surface and allowed to air dry. Using gloves, packaged and unpackaged tapes were rolled over their entire outside circumference onto the contaminated tiles to simulate cross contamination. Using new gloves, the packaged tapes were then removed from their package with minimum contact. All cross-contaminated tape rolls were placed in phosphate-buffered water and mixed in a vortexer for bacterial recovery procedures. Serial dilutions were plated on appropriate media for bacterial enumeration. The average log10 colony-forming unit (CFU) recovery was measured for comparison. We used 4 types of tapes in this study (3M Micropore S Surgical Tape, 3M Medipore H Soft Cloth Surgical Tape, 3M Durapore Surgical Tape, and 3M Transpore White Surgical Tape). We used 4 different microorganisms as inoculates: *Staphylococcus aureus* (methicillin-resistant), *Enterococcus faecium* (vancomycin-resistant), *Klebsiella pneumoniae* (carbapenem-resistant), and *Clostridium difficile* (spore). Each test (tape and bacteria combination) was done in 3 or 6 replicates; each bacterial enumeration was the average of duplicate plates. The detection limit for this method is 8 CFU per sample, which is equivalent to 0.9 log10. **Results:** The results for all tapes tested showed a statistically significant lower mean log10 recovery of each of the microorganisms tested for packaged versus unpackaged tape (Figure 1). The mean differences of log10 recoveries from a packaged and unpackaged tape ranged from 2.51 log10 (for *S. aureus* on Micropore S) to 4.64 log10 (for *K. pneumoniae* on Medipore H). This is equivalent to 99%–99.99% cross-contamination protection from the 4 organisms tested. **Conclusions:** Individual packaging of medical tape rolls protects them from external contaminants. Even if the packaging becomes contaminated, the tape retrieved from the package will be significantly less contaminated than it would have been from exposure to the same contaminants without packaging.

**Funding:** 3M Company

**Disclosures:** None

**Figure 1.**